# Porcine Circovirus 2 Increases the Frequency of Transforming Growth Factor-β via the C35, S36 and V39 Amino Acids of the ORF4

**DOI:** 10.3390/v15071602

**Published:** 2023-07-22

**Authors:** Cheng Han, Weicheng Xu, Jianfang Wang, Xiaolin Hou, Shuanghai Zhou, Qinye Song, Xuewei Liu, Huanrong Li

**Affiliations:** 1 College of Animal Science and Technology, Beijing University of Agriculture, No. 7 Beinong Road, Beijing 102206, Chinawjfhlx@126.com (J.W.); hxlsx@163.com (X.H.);; 2 College of Veterinary Medicine, Hebei Agricultural University, Baoding 071000, China

**Keywords:** porcine circovirus 2, porcine intestinal epithelial cells, regulatory T cells, TGF-β

## Abstract

Porcine circovirus 2 (PCV2) is one of the most important endemic swine pathogens, inducing immunosuppression in pigs and predisposing them to secondary bacterial or viral infections. Our previous studies show that PCV2 infection stimulated pig intestinal epithelial cells (IPEC-J2) to produce the secretory transforming growth factor-β (TGF-β), which, in turn, caused CD4^+^ T cells to differentiate into regulatory T cells (T_regs_). This may be one of the key mechanisms by which PCV2 induces immunosuppression. Here, we attempt to identify the viral proteins that affect the TGF-β secretion, as well as the key amino acids that are primarily responsible for this occurrence. The three amino acids C35, S36 and V39 of the ORF4 protein are the key sites at which PCV2 induces a large amount of TGF-β production in IPEC-J2 and influences the frequency of T_regs_. This may elucidate the regulatory effect of PCV2 on the T_regs_ differentiation from the perspective of virus structure and intestinal epithelial cell interaction, laying a theoretical foundation for improving the molecular mechanism of PCV2-induced intestinal mucosal immunosuppression in piglets.

## 1. Introduction

Porcine circovirus 2 (PCV2) was discovered in 1998. The disease associated with PCV2 was initially referred to as postweaning multisystemic wasting syndrome (PMWS) [1]. In view of the fact that reproductive, digestive and respiratory abnormalities in pigs are related to PCV2 infection, this syndrome is collectively called porcine-circovirus-associated disease (PCVAD), resulting in huge losses in the global pig industry [2,3].

The replication of PCV2 is highly dependent on host cell processes [4,5]. During virus replication, PCV2 induces cytokine production, affecting host immune function and leading to cytokine imbalance and immunosuppression [6,7,8]. The number of dendritic cells (DCs), natural killer cells, T cells, CD4^+^ T and CD8^+^ T lymphocytes, as well as B cells, is downregulated in PCV2-infected piglets, whereas the number of monocytes and granulocytes is elevated [9]. Interleukin-2 (IL-2), Interleukin-4 (IL-4), Interleukin-6 (IL-6), Interleukin-10 (IL-10) and tumor necrosis factor-α (TNF-α) levels are elevated in peripheral blood mononuclear cells (PBMCs) from PCV2-infected piglets stimulated with concanavalin A [10]. High levels of interleukin-1beta (IL-1β), interleukin 8 (IL-8), IL-10, TNF-α, neutrophil chemokine-II, granulocyte colony-stimulating factor and monocyte chemotactic protein-1 are produced in porcine alveolar macrophages (PAM) infected with PCV2 [11,12].

Regulatory T cells (T_regs_) can be obtained from naïve T cells stimulated by TGF-β and IL-2; the resulting cells are referred to as induced T_regs_ (iT_regs_) when formed in vitro and are referred to as peripheral T_regs_ (pT_regs_) when generated in vivo [13]. As important mediators of immunological suppression, T_regs_ play crucial roles in many aspects of immune systems. Increased T_regs_ compromise the typical antiviral response and impair immunity [14,15,16]. Foxp3 expression induced by TGF-β can suppress the T cell clonal deletion and differentiation of pT_regs_, which differentiate further into fully suppressed effector T_regs_ in response to T cell antigen receptor (TCR) and cytokine signaling [15]. Dendritic cells infected with PCV2 can induce the production of T_regs_, which is related to the upregulation of TGF-β [17]. Intestinal porcine epithelial cells (IPEC-J2) infected with PCV2 can also upregulate the secretion of TGF-β, which can stimulate the differentiation of CD4^+^ T cells into T_regs_, indicating a potential immunosuppressive mechanism of intestinal mucosal immune caused by PCV2 infection [18]. However, the structural component of PCV2, which affects the generation of TGF-β, still is unclear.

In this study, we evaluate the viral proteins and amino acid sites associated with the enormous production of TGF-β and an increase in T_regs_ following the PCV2 infection of IPEC-J2. This may elucidate the regulatory effect of PCV2 on T_regs_ differentiation and lay a theoretical foundation for improving the molecular mechanism of PCV2-induced immunosuppression in piglets.

## 2. Materials and Methods

### 2.1. Animal, Cell and Virus

Intestinal porcine epithelial cells J2 (IPEC- J2) (from DSMZ, No. 701) were cultured in DMEM (Gibco, Grand Island, NY, USA) supplemented with 10% FBS (BI, Kibbutz Beit, Israel) at 37 °C in a 5% CO_2_ atmosphere (Thermo Fisher Scientific, Waltham, MA, USA). PCV2 strain SD/2008 (GenBank accession number GQ174519) was isolated and identified by Animal Infectious Disease Laboratory of Hebei Agricultural University.

Two thirty-day-old specific-pathogen-free (SPF) large white piglets (free of PCV1, PCV2, porcine respiratory and reproductive syndrome virus, classical swine fever virus, pseudorabies virus and *mycoplasma hyopneumoniae*) were purchased from Beijing Centre for SPF Swine Breeding and Management for separation of peripheral blood mononuclear cells (PBMCs).

### 2.2. Plasmid Construction and Transfection

Genes encoding ORF1, ORF2, ORF3, ORF4, ORF5 and ORF6 of PCV2 were amplified from PCV2 strain SD/2008 via PCR. The amplified fragment was ligated into the pcDNA3.1(+) vector (Addgene, Cambridge, MA, USA) with an N-terminal His tag, being named as *His-ORF1*, *His-ORF2*, *His-ORF3*, *His-ORF4*, *His-ORF5* and *His-ORF6*. Truncated versions of *ORF2* and *ORF4* (*His-ORF2* (1–116aa), *His-ORF2* (117–233aa), *His-ORF4* (1–29aa), *His-ORF4* (30–59aa), *His-ORF4* (1–20aa), *His-ORF4* (11–29aa), *His-ORF4* (30–49aa) and *His-ORF4* (40–59aa)) were subcloned from the *pcDNA3.1(+)-His-ORF2/4* plasmids. Site-directed mutagenesis was used to construct variants of the ORF4 protein containing single amino acid substitutions (Flag-ORF4N30A, Flag-ORF4V31A, Flag-ORF4T32A, Flag-ORF4G33A, Flag-ORF4C34A, Flag-ORF4C35A, Flag-ORF4S36A). Mutagenesis was performed using QuikChange II XL (Stratagene, La Jolla, CA, USA) according to the manufacturer’s instructions. Constructs were validated via DNA sequencing. All the primers are listed in Table 1. And IPEC-J2 were transfected with the constructed plasmids above using Lipofectamine 3000 according to the manufacturer’s instructions.

### 2.3. CD4^+^ T Cell Preparation

PBMCs were isolated via Lymphoprep (Haoyang Biological Manufacture, Tianjin, China) sedimentation and cultured in RPMI 1640 medium (Gibco, Grand Island, NY, USA) supplemented with 10 mM HEPES (pH 7.5; Sigma-Aldrich, Deisenhofen, Germany), 2 mM L-glutamine and 100 U/mL penicillin (Sigma). CD4^+^ T cells were isolated through positive selection using CD4 MicroBeads (Miltenyi, Bergisch Gladbach, Germany) (purity > 95%).

### 2.4. Co-Culture System

IPEC-J2 (2 × 10^6^ cells/well) were seeded and formed a monolayer on the bottom chambers of transwell system (Corning, NY, USA). IPEC-J2 were transfected with different *ORF* plasmid constructs, respectively, for 24 h and then co-cultured with isolated CD4^+^ T cells. CD4^+^ T cells were cultured at 3 × 10^6^ cells/well in the upper chamber, along with mAbs 1 μg/mL of anti-CD3 (Abcam, Cambridge, UK) and 1 μg/mL of anti-CD28 (Abcam, Cambridge, UK). After co-culturing for 48 h, CD4^+^ T cells in the upper chamber were collected for flow cytometric analysis. All cells were incubated in DMEM media (Gibco, Grand Island, NY, USA) containing 10% FBS at 37 °C with a 5% CO_2_ atmosphere.

For the co-culture of CD4^+^ T cells and IPEC-J2 infected with PCV2, the IPEC-J2 cells (2 × 10^6^ cells/well) were seeded on the bottom chambers of transwell system for 12 h. And IPEC-J2 were infected with PCV2 (MOI = 1) for 48 h. Then, in the upper chambers, fresh CD4^+^ T cells were added. After 48 h of co-culture, the cells from the upper chambers were analyzed via flow cytometry.

### 2.5. Flow Cytometry

Cells were stained with mouse anti-porcine CD4 monoclonal antibody, FITC (Southern Biotech, Birmingham, AL, USA) and Foxp3 monoclonal antibody, PE (eBioscience, San Diego, CA, USA) or with appropriate isotype controls. Attune NxT flow cytometer (Invitrogen, Carlsbad, CA, USA) and FlowJo software (Tree Star Inc., Stanford, CA, USA) were utilized for flow cytometric analysis.

### 2.6. Extraction of Total RNA and Quantitative RT-PCR

Cellular RNA was isolated using a Total RNA Kit I (Senkang, Beijing, China). According to the instructions provided by manufacturer, the RNA was reverse transcribed using a HiScript II 1st Strand cDNA Synthesis Kit (Vazyme, Nanjing, China), and UltraSYBR Mixture (Low ROX) was utilized for quantitative PCR (CW Bio., Beijing, China). The sequences of TGF-β1 and β-actin gene primers were followed by upstream primers 5′-GACGCCAAAATC-3′ and 5′-CTCATGAAGTGCGACGT-3′, downstream primers 5′-GACGACTGAGAGAGAGAA-3′ and 5′-GTGATCCTGCATCCGTC-3′, respectively. The data were given as fold changes in gene expression adjusted to β-actin and compared to the mock-infected control. Each reaction was performed in triplicate, and the data were expressed as means (M) and standard error of the mean (SEM).

### 2.7. ELISA

TGF-β concentrations in cell culture supernatant were determined using sandwich ELISA kits (R&D systems, Minneapolis, MN, USA) in accordance with the manufacturer’s protocols.

### 2.8. Immunofluorescence Staining and Confocal Microscopy

Each treatment of IPEC-J2 was fixed with 4% paraformaldehyde in PBS at 4 °C for 30 min. After 3 washes, the cells were permeabilized with 0.1% Triton X-100 for 15 min and then blocked with 5% bovine serum albumin (BSA) in PBS at 37 °C for 1 h. The cells were then treated at 37 °C for 1 h with the corresponding primary antibodies, such as mouse anti-flag (1:5000; Santa Cruz Biotechnology, Helena, MT, USA) or mouse anti-His (1:2500; Proteintech, Chicago, IL, USA), according to the manufacturer’s instructions. After washing with PBS, cells were incubated at 37 °C for 1 h in the dark with FITC-conjugated goat anti-mouse IgG (H-L) (1:200; Beyotime, Shanghai, China). DAPI (Invitrogen, Carlsbad, CA, USA) was used to stain nuclei at room temperature for 15 min. Using a Nikon A1 confocal microscope and the Axiovision automatic measuring application, the labeled cells were photographed and evaluated (Nikon A1; Nikon, Tokyo, Japan).

### 2.9. Statistical Analysis

All statistical analyses were performed using GraphPad Prism 7.0 software (Version X; La Jolla, CA, USA). The results were expressed as the mean ± SD. The significance of the differences among groups was determined via one-way or two-way analysis of variance. Differences with *p*-values < 0.05 or 0.01 were considered significant or extremely significant and designated with an asterisk (*) or two asterisks (**) in the figures, respectively. Unless indicated otherwise, the experiments were performed in triplicate (*n* = 3).

### 2.10. Ethics Statement

All animal experiments were performed in accordance with the National Guidelines for Housing and Care of Laboratory Animals (China) and with the agreement of Beijing University of Agriculture’s Institutional Animal Care and Ethics Committee (approval No. SYXK2019-0005). All piglets were housed in Beijing University of Agriculture’s animal facility (Beijing, China).

## 3. Results

### 3.1. Proteins Encoded by ORF2, ORF4 and ORF5 Induces the Production of TGF-β in IPEC-J2

**Figure 1:**
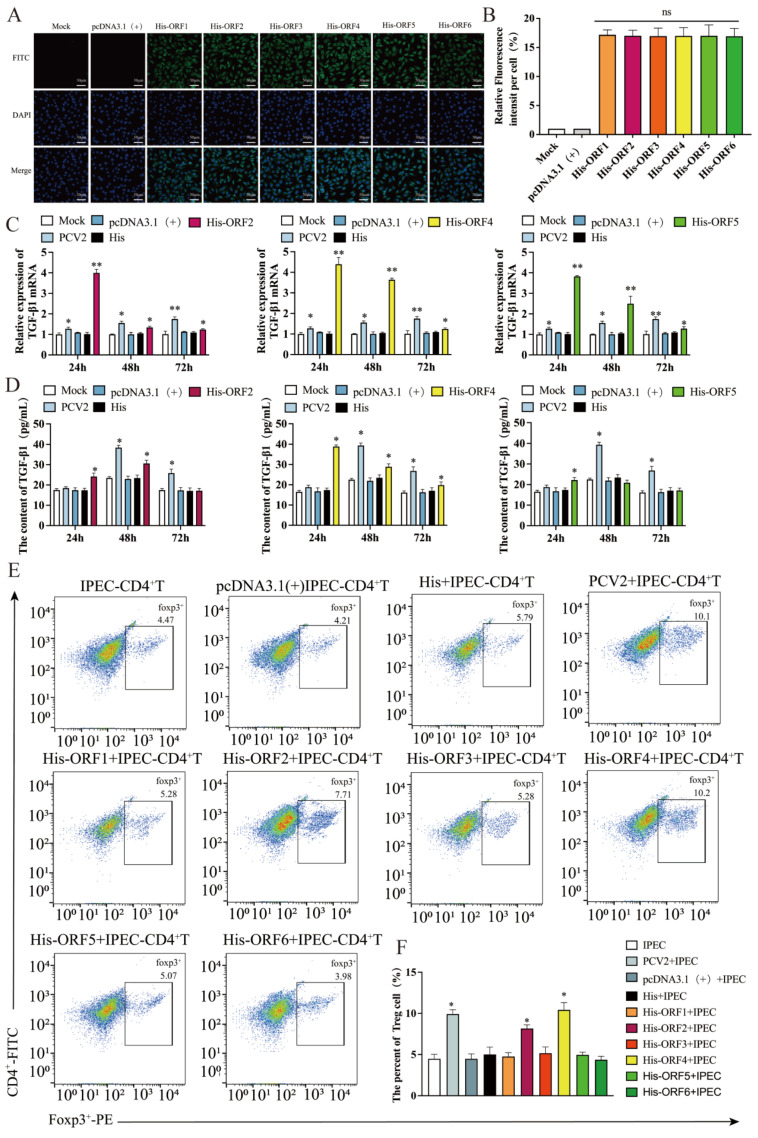
Regulation of TGF-β in IPEC by PCV2 ORFs and the impact of IPEC-J2 transfected with PCV2 ORFs on the T_reg_ subpopulations of CD4^+^ T cells. (**A**) Confocal microscopy of IPEC-J2 transfected with 1 μg of each plasmid encoding *His-ORF1*, *His-ORF2*, *His-ORF3*, *His-ORF4*, *His-ORF5* or *His-ORF6* for 24 h. Cells were fixed, reacted with anti-His antibody (green), and stained with DAPI (blue). (**B**) The expression statistics for each viral protein. A total of 1 μg of each plasmid encoding viral protein was transfected into IPEC-J2, respectively. (**C**) Relative expression levels of TGF-β mRNA determined via qPCR. (**D**) TGF-β level in cell supernatant detected via ELISA. (**E**,**F**) IPEC-J2 were transfected with 1 μg of each plasmid encoding viral protein for 24 h, and then co-cultured with CD4^+^ T cells. The percentage of T_regs_ was tested via flow cytometry after 48 h. All assays were performed in triplicate, with three technical repeats for each sample. **, *p* < 0.01; *, *p* < 0.05.

Our previous research has shown that PCV2 infection of IPEC-J2 can cause a substantial expression of TGF-β. In order to investigate the mechanism, we constructed six overexpression plasmids of viral proteins, known as *His-ORF1*, *His-ORF2*, *His-ORF3*, *His-ORF4*, *His-ORF5* and *His-ORF6*. The expression of *His* of different plasmids in IPEC-J2 was measured via immunofluorescence, indicating the expression of viral protein. The results demonstrate that the six plasmids could express the corresponding viral structural proteins successfully. In order to exclude the impact of expression differences of each structural protein on the experiment, the six plasmids were transfected into IPEC-J2 at the same dose of 1 μg, respectively, and their expression levels of *His* were similar (Figure 1A,B).

Then, 1 μg of each of the six plasmids were transfected into IPEC-J2, respectively, and the cells were collected at 24, 48 and 72 h post-transfection for determining the mRNA levels of the TGF-β via qRT-PCR. In the IPEC-J2 transfected with ORF2, ORF4 or ORF5 plasmid, the expression of TGF-β mRNA was considerably upregulated at 24, 48 and 72 h with a decreasing trend over time (Figure 1C). In contrast, the TGF-β mRNA expression did not change appreciably in the IPEC-J2 transfected with ORF1, ORF3 or ORF6 plasmid (data not shown). The levels of TGF-β in the different cell supernatant were evaluated via an ELISA at 24, 48 and 72 h after transfection of the six plasmids. Compared to the untreated IPEC-J2 (mock), TGF-β was considerably elevated in the supernatant of the IPEC-J2 transfected with ORF4 plasmid at 24 h, 48 h and 72 h (Figure 1D), while that in the supernatant of the IPEC-J2 transfected with ORF2 or ORF5 plasmid did not differ significantly at 72 h (Figure 1D). Similar to the results for the TGF-β mRNA, there were no significant differences in TGF-β in the supernatant of the IPEC-J2 transfected with His-ORF1, His-ORF3 or His-ORF6 (data not shown).

### 3.2. Overexpression of ORF2 and ORF4 in IPEC-J2 Enhanced the Frequency of T_regs_

Previous research has shown that PCV2-infected IPEC-J2 affects the differentiation of CD4^+^ T cells, and hence, the number of T_regs_ is increased. To investigate the effect of each coding protein of PCV2 on the differentiation of CD4^+^ T cells, the IPEC-J2 transfected, respectively, with six coding protein plasmids of the virus were co-cultured with purified CD4^+^ T cells from peripheral blood at 24 h post-transfection, while anti-CD3 and anti-CD28, the specific activating antibodies of CD4^+^ T cells, were added to maintain CD4^+^ T cells in a differentiated and activated state.

After co-culturing for 48h, the CD4^+^ T cells were collected, and their differentiation was observed using flow cytometry (1E). The flow cytometric analyses demonstrated that, similar to the results of the PCV2-infected cells, the IPEC-J2 transfected with *His-ORF2* or *His-ORF4* induced CD4^+^ T cell differentiation, leading to an increase in the frequency of T_regs_ (Figure 1F). In contrast, the IPEC-J2 transfected with *His-ORF1*, *His-ORF3*, *His-ORF5* or *His-ORF6* did not affect CD4^+^ T cell differentiation significantly (Figure 1F).

### 3.3. Overexpression of ORF4 (30–59aa) in IPEC-J2 Enhanced the Frequency of Tregs

**Figure 2:**
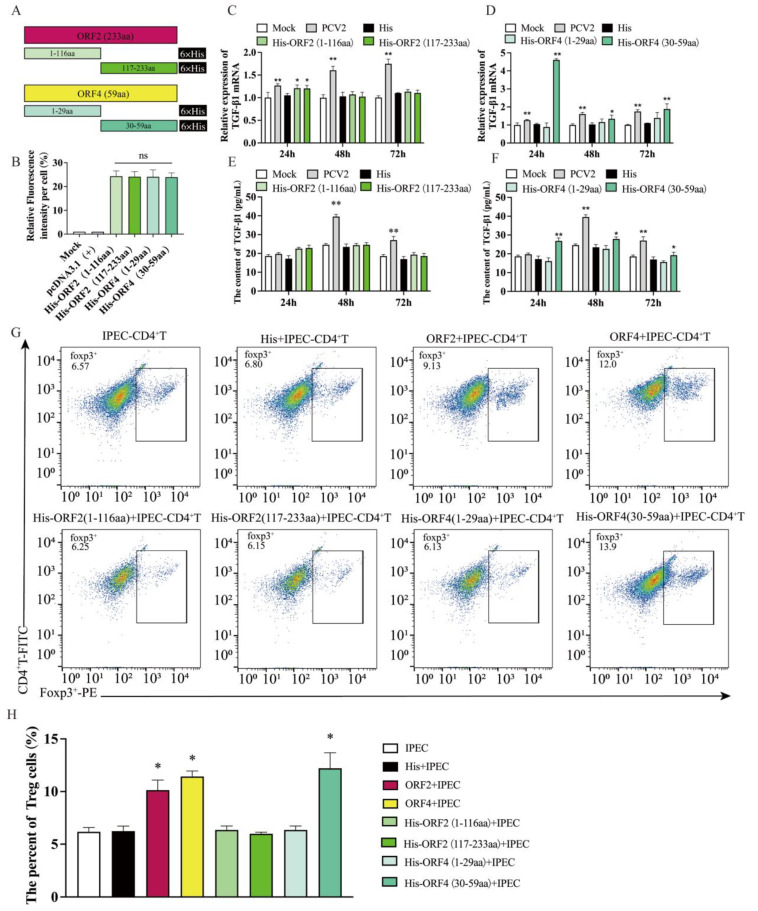
The impact of IPEC-J2 transfected with truncated *ORF2* and *ORF4* on the T_regs_ subpopulation of CD4^+^ T cell. (**A**) Schematic view showing regions of *ORF2* and *ORF4* expressed by truncation constructs. Numbers denote amino acid positions in ORF2 and ORF4. (**B**) The expression statistics for each viral protein; 1 μg of *His-ORF2* (1–116aa), *His-ORF2* (117–233aa), *His-ORF4* (1–29aa) and *His-ORF4* (30–59aa) was transfected into IPEC-J2, respectively. (**C**,**D**) Relative expression levels of TGF-β mRNA in cells determined via qPCR. (**E**,**F**) TGF-β in the supernatant of the cells detected via ELISA. (**G**,**H**) IPEC-J2 were transfected with 1 μg of plasmids for 24 h, respectively, and then co-cultured with CD4^+^ T cells. The percentage of T_regs_ was tested via flow cytometry after 48 h. All assays were performed in triplicate, with three technical repeats for each sample. **, *p* < 0.01; *, *p* < 0.05.

Given the above findings, it is evident that the *ORF2* and *ORF4* plasmids stimulated the secretion of large amounts of TGF-β by IPEC-J2 and upregulated the percentage of T_regs_, which was similar to the effect caused by the IPEC-J2 infected with PCV2. To determine the corresponding positions of the ORF2 and ORF4 exhibiting this effect, we truncated *ORF2* and *ORF4* separately again and produced four truncated mutant plasmids, namely *His-ORF2* (1–116aa), *His-ORF2* (117–233aa), *His-ORF4* (1–29aa) and *His-ORF4* (30–59aa) (Figure 2A). First, similarly, the immunofluorescence identified the expression of *His* in IPEC-J2 to exclude the impact of different truncated construct expression differences on the experiment (Figure 2B). Then, the four truncated plasmids were transfected into IPEC-J2, respectively, and the mRNA expression of TGF-β was evaluated at 24, 48 and 72 h after transfection. The results show that compared to the mock, the expression of TGF-β mRNA in the IPEC-J2 transfected with *His-ORF4* (30–59aa) was significantly upregulated at 24, 48 and 72 h, with statistical significance only at 24h in the IPEC-J2 transfected with *His-ORF2* (1–116aa) and *His-ORF2* (117–233aa) (Figure 2C,D), and the levels of TGF-β in the supernatant of IPEC-J2 transfected, respectively, with four truncated plasmid via ELISA show similar trends to those of TGF-β mRNA, and those of the IPEC-J2 transfected with *His-ORF4* (30–59aa) had significant differences at the three time points (Figure 2E,F).

The IPEC-J2 were transfected with four truncated plasmids, respectively, for 24 h, and then co-cultured with purified CD4^+^ T cells. After 48 h, the CD4^+^ T cells were collected for flow cytometry (2G). In response to the IPEC-J2 transfected with *His-ORF4* (30–59aa), the percentage of T_regs_ was increased significantly compared to the mock (Figure 2H). The IPEC-J2 transfected with *His-ORF2* (1–116aa), *His-ORF2* (117–233aa) or *His-ORF4* (1–29aa) had no impact on the CD4^+^ T cell differentiation (Figure 2H). The above results suggest that the 30–59aa position in the ORF4 of PCV2 is a key site that causes T cell differentiation, though ORF2 also has this effect, but it is not the main one.

### 3.4. The 30–39aa of ORF4 Altered the Frequency of T_regs_

**Figure 3:**
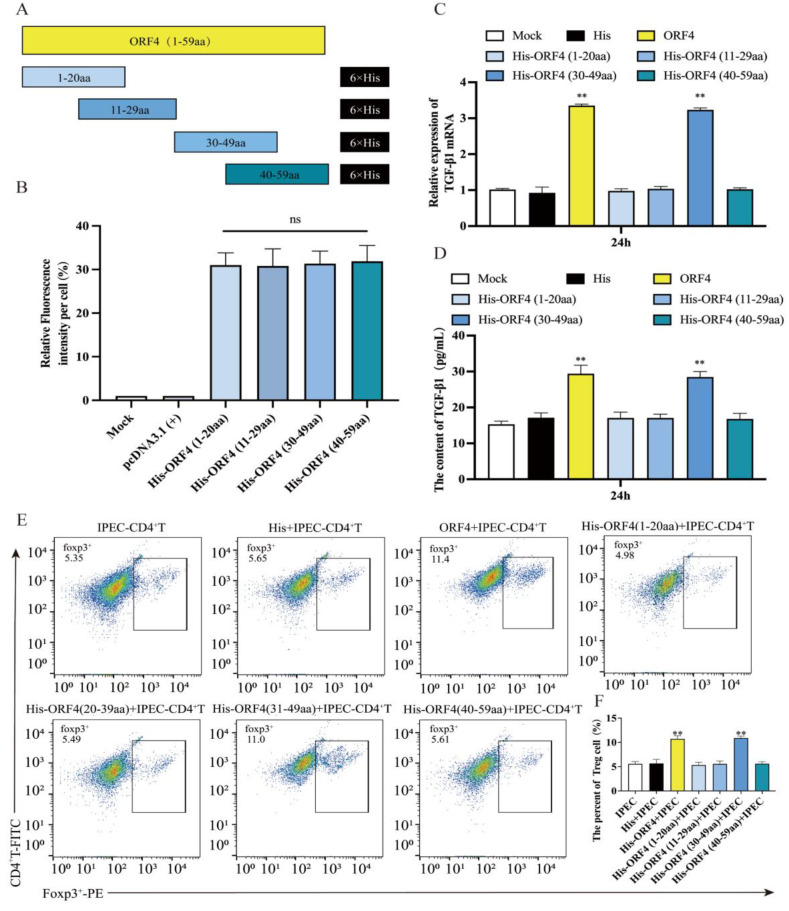
The impact of IPEC-J2 transfected with truncated ORF4 on the T_regs_ subpopulation of CD4^+^ T cell. (**A**) Schematic view showing regions of ORF4 expressed by truncation constructs. Numbers denote amino acid positions in ORF4. (**B**) The expression statistics for *His* of each truncated plasmid; 1 μg of each plasmid was transfected into IPEC-J2, respectively. (**C**) Relative expression levels of TGF-β mRNA in cells determined via qPCR. (**D**) TGF-β in the supernatant of the cells detected via ELISA. (**E**,**F**) IPEC-J2 were transfected with 1 μg of plasmids for 24 h, respectively, and then co-cultured with CD4^+^ T cells. The percentage of T_regs_ was tested via flow cytometry after 48 h. All assays were performed in triplicate, with three technical repeats for each sample. **, *p* < 0.01.

To investigate the functional amino sites of the PCV2 *ORF4*-encoded protein, we constructed four additional *ORF4*-truncated plasmids, namely *His-ORF4* (1–20aa), *His-ORF4* (11–29aa), *His-ORF4* (30–49aa) and *His-ORF4* (40–59aa) (Figure 3A). The immunofluorescence showed the expression of *His* in IPEC-J2 transfected with the four plasmids (Figure 3B).

At 24 h after transfection, the mRNA and protein levels of the TGF-β were increased in the IPEC-J2 transfected with *His-ORF4* (30–49aa) (Figure 3C,D). After, the IPEC-J2 were transfected with four plasmids, respectively, for 24 h, and then co-cultured with activated CD4^+^ T cells for 48 h. The flow cytometry demonstrated that the IPEC-J2 transfected with *His-ORF4* (30–49aa) promoted the differentiation of CD4^+^ T cells towards T_regs_, while the other three truncators had no such effect (Figure 3E,F). Due to the presence of overlapping 40–49aa fragments between the ORF4 40–59aa and ORF4 30–49aa, and the fact that the IPEC-J2 transfected with *His-ORF4* (40–59aa) did not affect the percentage of T_regs_, it can be inferred that the 30–39aa domain of the PCV2 ORF4 is a key region causing T cell differentiation.

### 3.5. C35, S36 and V39 Were Key Loci for ORF4-Induced Changes of T_regs_ Subpopulation

**Figure 4:**
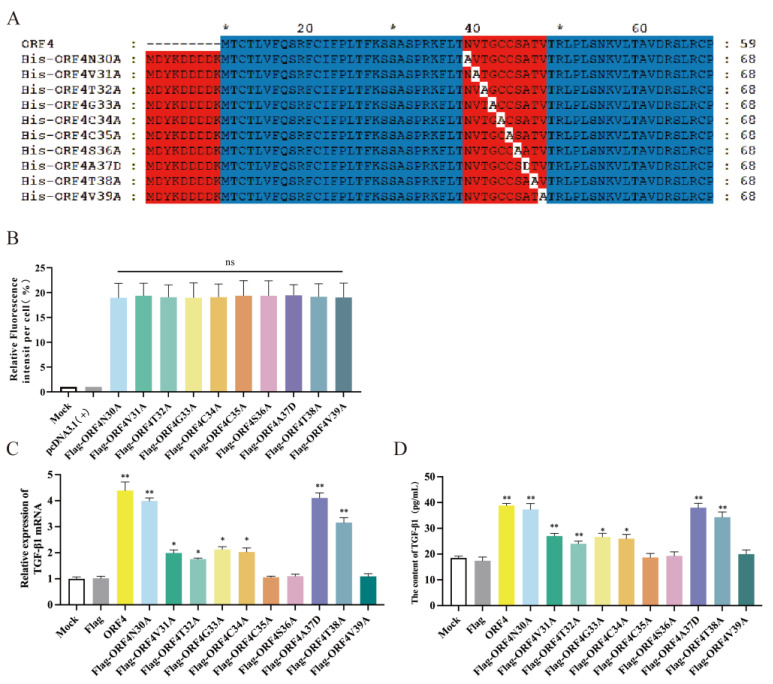
The effect of ORF4 mutants on TGF-β in IPEC-J2. (**A**) Schematic view showing regions of ORF4 expressed by mutant constructs. Numbers denote amino acid positions in ORF4. (**B**) The expression statistics for *Flag of* each mutant protein; 1 μg of each plasmid was transfected into IPEC-J2, respectively. (**C**) Relative expression levels of TGF -β mRNA in cells determined via qPCR. (**D**) TGF-β in the supernatant of the cells detected via ELISA. All assays were performed in triplicate, with three technical repeats for each sample. **, *p* < 0.01; *, *p* < 0.05.

Given that we located the functional position of ORF4 to 30–39aa, we separately mutated each of the ten amino acids to obtain ten mutant plasmids, namely *Flag-ORF4N30A*, *Flag-ORF4V31A*, *Flag-ORF4T32A*, *Flag-ORF4G33A*, *Flag-ORF4C34A*, *Flag-ORF4C35A*, *Flag-ORF4S36A*, *Flag-ORF4A37D*, *Flag-ORF4T38A* and *Flag-ORF4V39A* (Figure 4A). The immunofluorescence showed that the *Flag* of the ten mutant plasmids were expressed at the same level (Figure 4B). We investigated whether the ten mutant plasmids affected the mRNA expression and secretion of TGF-β in IPEC-J2. The findings demonstrate that in comparison to the mock, the expression of mRNA and protein of TGF-β from the IPEC-J2 transfected with the seven mutant plasmids were significantly upregulated, except there were little changes for the IPEC-J2 transfected with *Flag-ORF4C35A*, *Flag-ORF4S36A* or *Flag-ORF4V39A* (Figure 4C,D), indicating that the *ORF4*-encoded proteins 30–39aa, specifically C35, S36 and V39, could be crucial for the PCV2-infected IPEC-J2 to express TGF-β.

**Figure 5:**
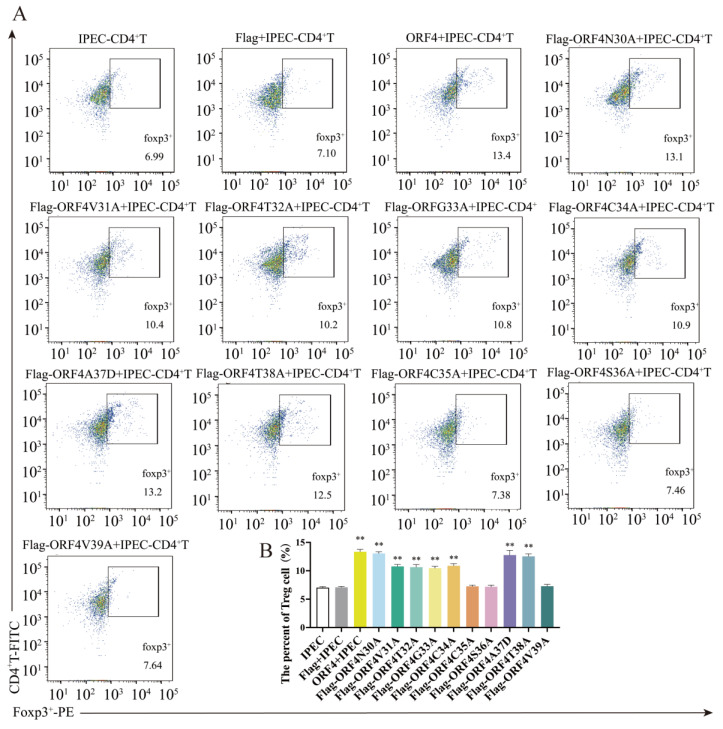
The impact of IPEC-J2 transfected with ORF4 mutants on the T_regs_ subpopulation of CD4^+^ T cell. IPEC-J2 were transfected with 1 μg of *Flag-ORF4N30A*, *Flag-ORF4V31A*, *Flag-ORF4T32A*, *Flag-ORF4G33A*, *Flag-ORF4C34A*, *Flag-ORF4C35A*, *Flag-ORF4S36A*, *Flag-ORF4A37D*, *Flag-ORF4T38A* or *Flag-ORF4V39A* for 24 h, and then co-cultured with CD4^+^ T cells. The percentage of T_regs_ was tested via flow cytometry after 48 h (**A**,**B**). All assays were performed in triplicate, with three technical repeats for each sample. **, *p* < 0.01.

Subsequently, the IPEC-J2 were transfected with ten mutant plasmids, respectively, for 24 h and then co-cultured with activated CD4^+^ T cells. After 48 h, the IPEC-J2 transfected with mutant *Flag-ORF4N30A*, *Flag-ORF4V31A*, *Flag-ORF4T32A*, *Flag-ORF4G33A*, *Flag-ORF4C34A*, *Flag-ORF4A37D* or *Flag-ORF4T38A* could still trigger CD4^+^ T cell differentiation towards T_regs_. Contrarily, the IPEC-J2 transfected with *Flag-ORF4C35A*, *Flag-ORF4S36A* or *Flag-ORF4V39A* did not show a significant differentiation of CD4^+^ T cells into T_regs_. In other words, the mutations in these three amino acids almost completely impeded the effect of ORF4 on CD4^+^ T cells differentiating into T_regs_ (Figure 5A,B).

In conclusion, the upregulation of TGF-β in the IPEC-J2 infected with PCV2 can promote the differentiation of CD4^+^ T cells into T_regs_. The expression of *ORF4* enhanced the frequency of T_regs_. Of the ORF4s, 30–59aa plays a critical role in altering the CD4^+^ T cell differentiation. The truncation and mutation of the protein encoded by *ORF4* revealed that the C35, S36 and V3 of PCV2 ORF4 were the key amino acid sites of PCV2, which induced the changes of the T_regs_ subset.

## 4. Discussion

PCV2 is the main pathogen responsible for porcine-circovirus-associated disease (PCVAD) [2]. The virus affects the lymphoid tissue preferentially, resulting in lymphoid tissue loss and immunosuppression in pigs. Immune stimulation or co-infection with other pathogens aggravates the condition [19]. The majority of PCVAD is a post-weaning multisystem failure syndrome (PMWS) that primarily affects pigs aged 5 to 18 weeks. Progressive weight loss, shortness of breath, anemia, diarrhea and jaundice are PMWS symptoms [20]. Pigs with PMWS have microscopic lymphoid tissue lesions characterized by the depletion of lymphocytes in the follicular and form regions and macrophage infiltration [21]. PCV2 can also cause enteritis, leading to immune disorders in the intestinal mucosa of infected pigs [22,23]. Intestinal epithelial cells can regulate CD4^+^ T cells and play an important role in the immune response of intestinal mucosal defense [24]. IPEC-J2, a type of intestinal mucosal epithelial cells derived from the middle ileum of newborn piglets, which can express related immune factors such as IL-6, TGF- β, IL-8, pIgR, secretory leukocyte protease inhibitors (SLPI), etc., are ideal cell models for studying intestinal mucosal immunity [25,26]. Our previous studies have shown that PCV2 can upregulate the secretion of TGF-β by IPEC-J2, promoting the differentiation of CD4^+^ T cells into T_regs_, which may be a new mechanism of PCV2’s immunosuppression of pigs [18].

Numerous cell types, including epithelial, endothelial, hematopoietic and immunological cells, are severely inhibited by TGF-β in terms of cell proliferation [27]. Additionally, TGF-β and T_regs_ are required for the inhibition of vertebrate auto-reactive T cells, as well as for the maintenance of autoimmune tolerance. It has also been reported that HIV plays a significant role in immunosuppression. High levels of TGF-β have been found in many HIV-positive patients, and T cells exhibit proliferative abnormalities early on [28,29].

We used IPEC-J2 as an in vitro model to investigate which viral proteins of PCV2 can increase the expression of TGF-β and influence the differentiation of CD4^+^ T cells into T_regs_. Six of the eleven predicted open reading frames in the PCV2 genome were identified, which can encode different viral proteins and perform different functions [30,31]. The ORF1 gene expresses the Rep protein, which is believed to be a crucial immunogenic protein of PCV2 [32,33]. The protein encoded by the ORF2 gene is the PCV2 capsid protein (Cap) [34]. ORF3 mainly encodes nucleoprotein, which can effectively cause cell apoptosis (e.g., PK15, PBMCs and lymphocytes) [35,36]. ORF4 can decrease caspase-3 and caspase-8 activity and regulate CD4^+^ T and CD8^+^ T cells to influence the host immune system [37]. ORF5 may maintain chronic PCV2 infection by modulating the NF-κB signaling pathway [38]. ORF6 expression alone can lead to a large rise in the expression of TNF-α, IL-10, IL-12 and IL-13 [32].

In this experiment, six viral protein expression plasmids of PCV2 were constructed, and their roles were investigated. The results indicate that the proteins encoded by *ORF2*, *ORF4* and *ORF5* may cause a high expression level of TGF-β in IPEC-J2. Among them, the *ORF4*-encoded viral protein had the most significant effect. And the IPEC-J2 transfected with *ORF2* or *ORF4* recombinant plasmids also promoted the differentiation of CD4^+^ T cells into T_regs_, while the IPEC-J2 transfected with ORF5 plasmid had little such effect.

To further investigate the major functional positions of the viral protein that promotes T cell differentiation, we truncated the *ORF2*-encoded protein and the *ORF4*-encoded protein, respectively. It was discovered that the 30–39aa polypeptide of the *ORF4*-encoded protein induced IPEC-J2 to express TGF-β in large amounts and could cause CD4^+^ T cells to differentiate into T_regs_ in a co-culture system, resulting in a larger proportion of T_regs_. In co-culture tests with CD4^+^ T cells, none of the *ORF2*-encoded protein truncates could enhance the proportion of T_regs_. The speculated reason for this may be that there was not enough time and an insufficient amount of TGF-β to affect the differentiation of CD4^+^ T cells into T_regs_, as truncated *ORF2* encoded proteins could enhance the TGF-β expression in IPEC-J2 with no statistical significance compared to the mock. However, the 30–39aa peptide of ORF4 could continuously induce high levels of TGF-β expression in IPEC-J2, thus inducing a high proportion of T_regs_ in co-cultured CD4^+^ T cells.

Ten-point mutant plasmids of *ORF4* were generated by continuing to alter each of the amino acids of ORF4 30–39aa that were initially targeted, and two typically inactive amino acids, alanine and aspartate, were chosen as mutants. Further research demonstrated that mutations in *C35*, *S36* or *V39* of *ORF4* greatly impacted the induction of ORF4 on IPEC-J2 following mutation, which meant that the TGF-β expression in transfected IPEC-J2 and the proportion of T_regs_ in the co-culture did not significantly increase. This indicates that C35, S36 and V39 play important roles in the induction function of ORF4, which may be crucial for the production of TGF-β in IPEC-J2 infected with PCV2 and changes in the T_regs_ subpopulation. Given that PCV2 has five genotypes, such as PCV2a, PCV2b, PCV2c, PCV2d and PCV2e [39,40], we also analyzed the ORF4 amino acid sequences of the different genotypes of PCV2 and discovered that all of the ORF4 sequences were highly conserved and similar, except for the two amino acid changes in the ORF4 of PCV2e. This means that the impact of the ORF4 of all PCV2 genotypes on the TGF-β in IPEC-J2 and on CD4^+^ T cells should be the same or similar.

In the previous study, we determined that PCV2-infected IPEC-J2 activated NF-κB to stimulate the synthesis of TGF-β, which enhanced the differentiation of CD4^+^ T cells into T_regs_ through the activation of ERK in CD4^+^ T cells. It is worth further researching the PCV2 infectious clone that is used to generate mutated viruses in a safe manner to confirm the function of these key amino acids and whether the key amino acid sites of ORF4 found in this study determine the role of PCV2 in immunosuppression.

In conclusion, the three amino acids C35, S36 and V39 of the ORF4 protein are the key amino acid sites at which PCV2 induces a large production of TGF-β in IPEC-J2 and influences the T_regs_ subpopulation. This study will help to create an understanding of the pathogenic mechanism of PCV2, especially in the intestinal mucosal immunosuppression caused by PCV2 from the interaction between its protein structure and intestinal epithelial cells.

## Figures and Tables

**Table 1:** The primers of PCR.

Genes	Primer Sequence (5′-3′)
*ORF1*	Forward: CTAGCGTTTAAACTTAAGCTTATGCCCAGTAAGAAGAATGGAAGA Reverse: ATGGTGGCGACCGGTGGATCCTCAATGATGATGATGATGATGGTAA
*ORF2*	Forward: CTAGCGTTTAAACTTAAGCTTATGACGTATCCAAGGAGGCGT Reverse: ATGGTGGCGACCGGTGGATCCTCAATGATGATGATGATGATGCTTA
*ORF3*	Forward: CTAGCGTTTAAACTTAAGCTTATGGTAACCATCCCACCACTTG Reverse: ATGGTGGCGACCGGTGGATCCTCAATGATGATGATGATGATGTGTCC
*ORF4*	Forward: CTAGCGTTTAAACTTAAGCTTATGCCCAGTAAGAAGAATGGAAGA Reverse: ATGGTGGCGACCGGTGGATCCCTAATGATGATGATGATGATGCAAAC
*ORF5*	Forward: CTAGCGTTTAAACTTAAGCTTATGGTTTTTATTATTCATTTAGGGTTTAAC Reverse: ATGGTGGCGACCGGTGGATCCCTAATGATGATGATGATGATGCAAAC
*ORF6*	Forward: CTAGCGTTTAAACTTAAGCTTATGGCATCTTCAACACCCGC Reverse: ATGGTGGCGACCGGTGGATCCTCAATGATGATGATGATGATGTGTCC
*ORF2 1–116A*	Forward: CACACTGGACTAGTGGATCCATGCATCATCATCATCATCATACGTATCCAAGGAGG Reverse: CAGCGGTTTAAACTTAAGCTTTCACCTGTCACCCTGGGTGAGT
*ORF2 117–233A*	Forward: CACACTGGACTAGTGGATCCATGCATCATCATCATCATCATAGGAGTGGGCTCCAC Reverse: CAGCGGTTTAAACTTAAGCTTTCAAGGGTTAAGTGGGGGGT
*ORF4 1–29A*	Forward: CCACACTGGACTAGTGGATCCATGCATCATCATCATCATCATACCTGCACCCTGGTG Reverse: CAGCGGTTTAAACTTAAGCTTTCAGTTGGTCAGGAATTTGCG
*ORF4 30–59A*	Forward: CCACACTGGACTAGTGGATCCATGCATCATCATCATCATCATGTGACCGGCTGC Reverse: CAGCGGTTTAAACTTAAGCTTTCATGGGCACCTCAGGGAG
*ORF4 1–20A*	Forward: TAGCGTTTAAACTTAAGCTTATGCATCATCATCATCATCATACCTGCACCCTGGTG Reverse: CCACACTGGACTAGTGGATCCTCAGCTCTTGAAGGTCAGGGG
*ORF4 11–29A*	Forward: CTAGCGTTTAAACTTAAGCTTATGCATCATCATCATCATCATTGCATCTTCCC Reverse: CCACACTGGACTAGTGGATCCTCAGTTGGTCAGGAATTTGCG
*ORF4 30–49A*	Forward: CTAGCGTTTAAACTTAAGCTTATGCATCATCATCATCATCATGTGACCGGCTGC Reverse: CCACACTGGACTAGTGGATCCTCATGGGCACCTCAGGGAG
*ORF4 40–59A*	Forward: CTAGCGTTTAAACTTAAGCTTATGCATCATCATCATCATCATGTGACCGGCTG Reverse: CCACACTGGACTAGTGGATCCTCACAGCACTTTGTTGCTCAGG
*ORF4N30A*	Forward: ATGGATTACAAGGACGACGATGACAAGATGACCTGCAC Reverse: GCCGGTGGCGTTGGT Forward: GGTCACGGCGGTCAG Reverse: TGATTCTCATCAAGCAGGTCTCC
*ORF4V31A*	Forward: ATGGATTACAAGGACGACGATGACAAGATGACCTGCAC Reverse: GCCGGTGGCGTTGGT Forward: ACCAACGCCACCGGC Reverse: TGATTCTCATCAAGCAGGTCTCC
*ORF4T32A*	Forward: ATGGATTACAAGGACGACGATGACAAGATGACCTGCAC Reverse: GCAGCCGGCCACGTT Forward: AACGTGGCCGGCTGC Reverse: TGATTCTCATCAAGCAGGTCTCC
*ORF4G33A*	Forward: ATGGATTACAAGGACGACGATGACAAGATGACCTGCAC Reverse: GCAGCAGGCGGTCAC Forward: GTGACCGCCTGCTGC Reverse: TGATTCTCATCAAGCAGGTCTCC
*ORF4V34A*	Forward: ATGGATTACAAGGACGACGATGACAAGATGACCTGCAC Reverse: GGAGCAGGCGCCGGT Forward: ACCGGCGCCTGCTCC Reverse: TGATTCTCATCAAGCAGGTCTCC
*ORF4V35A*	Forward: ATGGATTACAAGGACGACGATGACAAGATGACCTGCAC Reverse: GGCGGAGGCGCAGCC Forward: GGCTGCGCCTCCGCC Reverse: TGATTCTCATCAAGCAGGTCTCC
*ORF4S36A*	Forward: ATGGATTACAAGGACGACGATGACAAGATGACCTGCAC Reverse: GGTGGCGGCGCAGCA Forward: TGCTGCGCCGCCACC Reverse: TGATTCTCATCAAGCAGGTCTCC
*ORF4A37D*	Forward: ATGGATTACAAGGACGACGATGACAAGATGACCTGCAC Reverse: CACGGTGTCGGAGCA Forward: TGCTCCGACACCGTG Reverse: TGATTCTCATCAAGCAGGTCTCC
*ORF4T38A*	Forward: ATGGATTACAAGGACGACGATGACAAGATGACCTGCAC Reverse: GGTCACGGCGGCGGA Forward: TCCGCCgccGTGACC Reverse: TGATTCTCATCAAGCAGGTCTCC
*ORF4V39A*	Forward: ATGGATTACAAGGACGACGATGACAAGATGACCTGCAC Reverse: CCTGGTGGCGGTGGC Forward: GCCACCGCCACCAGG Reverse: TGATTCTCATCAAGCAGGTCTCC

## Data Availability

The datasets generated and analyzed during the current study are available from the corresponding author on reasonable request.

## References

[1] Allan G.M., Mcneilly F., Kennedy S., Daft B., Clarke E.G., Ellis J.A., Haines D.M., Meehan B.M., Adair B.M. (1998). Isolation of porcine circovirus-like viruses from pigs with a wasting disease in the USA and Europe. J. Vet. Diagn. Investig..

[2] Opriessnig T., Meng X.J., Halbur P.G. (2007). Porcine circovirus type 2 associated disease: Update on current terminology, clinical manifestations, pathogenesis, diagnosis, and intervention strategies. J. Vet. Diagn. Investig..

[3] Segalés J. (2012). Porcine circovirus type 2 (PCV2) infections: Clinical signs, pathology and laboratory diagnosis. Virus Res..

[4] Mankertz A. (2012). Molecular interactions of porcine circoviruses type 1 and type 2 with its host. Virus Res..

[5] Cheung A.K. (2012). Porcine circovirus: Transcription and DNA replication. Virus Res..

[6] Ramamoorthy S., Meng X.J. (2009). Porcine circoviruses: A minuscule yet mammoth paradox. Anim. Health Res. Rev..

[7] Zhai N., Liu K., Li H., Liu Z., Wang H., Korolchuk V.I., Carroll B., Pan C., Gan F., Huang K. (2019). PCV2 replication promoted by oxidative stress is dependent on the regulation of autophagy on apoptosis. Vet. Res..

[8] Unterweger C., Brunthaler R., Auer A., Fux R., Weissenbacher-Lang C., Ladinig A. (2021). Reconsideration of the diagnostic criteria required for PCV2 reproductive disease. Vet. J..

[9] Darwich L., Segalés J., Domingo M., Mateu E. (2002). Changes in CD4 (^+^), CD8 (^+^), CD4 (^+^) CD8 (^+^), and immunoglobulin M-positive peripheral blood mononuclear cells of postweaning multisystemic wasting syndrome-affected pigs and age-matched uninfected wasted and healthy pigs correlate with lesions and porcine circovirus type 2 load in lymphoid tissues. Clin. Diagn. Lab. Immunol..

[10] Shi K.C., Guo X., Ge X.N., Liu Q., Yang H.-C. (2010). Cytokine mRNA expression profiles in peripheral blood mononuclear cells from piglets experimentally co-infected with porcine reproductive and respiratory syndrome virus and porcine circovirus type 2. Vet. Microbiol..

[11] Sun N., Sun P., Lv H., Sun Y., Guo J., Wang Z., Luo T., Wang S., Li H. (2016). Matrine displayed antiviral activity in porcine alveolar macrophages co-infected by porcine reproductive and respiratory syndrome virus and porcine circovirus type 2. Sci. Rep..

[12] Han J., Zhang S., Zhang Y., Chen M., Lv Y. (2017). Porcine circovirus type 2 increases interleukin-1beta and interleukin-10 production via the MyD88-NF-kappa B signaling pathway in porcine alveolar macrophages in vitro. J. Vet. Sci..

[13] Kanamori M., Nakatsukasa H., Okada M., Lu Q., Yoshimura A. (2016). Induced Regulatory T Cells: Their Development, Stability, and Applications. Trends Immunol..

[14] Sakaguchi S., Miyara M., Costantino C.M., Hafler D.A. (2010). FOXP3^+^ regulatory T cells in the human immune system. Nat. Rev. Immunol..

[15] Savage P.A., Klawon D.E.J., Miller C.H. (2020). Regulatory T Cell Development. Annu. Rev. Immunol..

[16] Liu C., Zeng X., Yu S., Ren L., Sun X., Long Y., Wang X., Lu S., Song Y., Sun X.-H. (2021). Up-regulated DNA-binding inhibitor Id3 promotes differentiation of regulatory T cell to influence antiviral immunity in chronic hepatitis B virus infection. Life Sci..

[17] Cecere T.E., Meng X.J., Pelzer K., Todd S., Beach N., Ni Y., LeRoith T. (2012). Co-infection of porcine dendritic cells with porcine circovirus type 2a (PCV2a) and genotype II porcine reproductive and respiratory syndrome virus (PRRSV) induces CD4(^+^)CD25(^+^)FoxP3(^+^) T cells in vitro. Vet. Microbiol..

[18] Liu X., Wang Y., Han C., Li Q., Hou X., Song Q., Zhou S., Li H. (2022). TGF-β from the Porcine Intestinal Cell Line IPEC-J2 Induced by Porcine Circovirus 2 Increases the Frequency of Treg Cells via the Activation of ERK (in CD4(^+^) T Cells) and NF-κB (in IPEC-J2). Viruses.

[19] Sun N., Zhang H., Sun P., Khan A., Guo J., Zheng X., Sun Y., Fan K., Yin W., Li H. (2020). Matrine exhibits antiviral activity in a PRRSV/PCV2 co-infected mouse model. Phytomedicine.

[20] Kang L., Wahaab A., Shi K., Mustafa B.E., Zhang Y., Zhang J., Li Z., Qiu Y., Li B., Liu K. (2022). Molecular Epidemic Characteristics and Genetic Evolution of Porcine Circovirus Type 2 (PCV2) in Swine Herds of Shanghai, China. Viruses.

[21] Marruchella G., Valbonetti L., Bernabò N., Ligios C. (2017). Depletion of follicular dendritic cells in tonsils collected from PMWS-affected pigs. Arch. Virol..

[22] Shi F., Li Q., Zou Z., Wang Y., Hou X., Zhang Y., Song Q., Zhou S., Li H. (2020). The changes of immune-related molecules within the ileal mucosa of piglets infected with porcine circovirus type 2. J. Vet. Sci..

[23] Baró J., Segalés J., Martínez J. (2015). Porcine circovirus type 2 (PCV2) enteric disease: An independent condition or part of the systemic disease?. Vet. Microbiol..

[24] Lu J.T., Xu A.T., Shen J., Ran Z.H. (2017). Crosstalk between intestinal epithelial cell and adaptive immune cell in intestinal mucosal immunity. J. Gastroenterol. Hepatol..

[25] Mariani V., Palermo S., Fiorentini S., Lanubile A., Giuffra E. (2009). Gene expression study of two widely used pig intestinal epithelial cell lines: IPEC-J2 and IPI-2I. Vet. Immunol. Immunopathol..

[26] Lan D., Tang C., Yue H., Sun H., Cui L., Hua X., Li J. (2013). Microarray analysis of differentially expressed transcripts in porcine intestinal epithelial cells (IPEC-J2) infected with porcine sapelovirus as a model to study innate immune responses to enteric viruses. Arch. Virol..

[27] Zhang Y., Alexander P.B., Wang X.F. (2017). TGF-β Family Signaling in the Control of Cell Proliferation and Survival. Cold Spring Harb. Perspect. Biol..

[28] Dickinson M., Kliszczak A.E., Giannoulatou E., Peppa D., Pellegrino P., Williams I., Drakesmith H., Borrow P. (2020). Dynamics of Transforming Growth Factor (TGF)-β Superfamily Cytokine Induction During HIV-1 Infection Are Distinct from Other Innate Cytokines. Front. Immunol..

[29] Reinhold D., Wrenger S., Kähne T., Ansorge S. (1999). HIV-1 Tat: Immunosuppression via TGF-beta1 induction. Immunol. Today.

[30] Mankertz A., Hillenbrand B. (2001). Replication of porcine circovirus type 1 requires two proteins encoded by the viral rep gene. Virology.

[31] Cheung A.K. (2003). Comparative analysis of the transcriptional patterns of pathogenic and nonpathogenic porcine circoviruses. Virology.

[32] Fort M., Sibila M., Nofrarías M., Pérez-Martín E., Olvera A., Mateu E., Segalés J. (2010). Porcine circovirus type 2 (PCV2) Cap and Rep proteins are involved in the development of cell-mediated immunity upon PCV2 infection. Vet. Immunol. Immunopathol..

[33] Nawagitgul P., Morozov I., Bolin S.R., Harms P.A., Sorden S.D., Paul P.S. (2000). Open reading frame 2 of porcine circovirus type 2 encodes a major capsid protein. J. Gen. Virol..

[34] He J., Cao J., Zhou N., Jin Y., Wu J., Zhou J. (2013). Identification and functional analysis of the novel ORF4 protein encoded by porcine circovirus type 2. J. Virol..

[35] Liu J., Chen I., Du Q., Chua H., Kwang J. (2006). The ORF3 protein of porcine circovirus type 2 is involved in viral pathogenesis in vivo. J. Virol..

[36] Liu J., Zhu Y., Chen I., Lau J., He F., Lau A., Wang Z., Karuppannan A.K., Kwang J. (2007). The ORF3 protein of porcine circovirus type 2 interacts with porcine ubiquitin E3 ligase Pirh2 and facilitates p53 expression in viral infection. J. Virol..

[37] Lv Q., Guo K., Xu H., Wang T., Zhang Y. (2015). Identification of Putative ORF5 Protein of Porcine Circovirus Type 2 and Functional Analysis of GFP-Fused ORF5 Protein. PLoS ONE.

[38] Franzo G., Segalés J. (2018). Porcine circovirus 2 (PCV-2) genotype update and proposal of a new genotyping methodology. PLoS ONE.

[39] Jang G., Yoo H., Kim Y., Yang K., Lee C. (2021). Genetic and phylogenetic analysis of porcine circovirus type 2 on Jeju Island, South Korea 2019–2020: Evidence of a novel intergenotypic recombinant. Arch. Virol..

[40] Rajkhowa T.K., Lalnunthanga P., Rao P.L., Subbiah M., Lalrohlua B. (2021). Emergence of porcine circovirus 2g (PCV2g) and evidence for recombination between genotypes 2g, 2b and 2d among field isolates from non-vaccinated pigs in Mizoram, India. Infect. Genet. Evol..

